# The surgical strategy of hormonally active primary cardiac paraganglioma sarcoma: A case report

**DOI:** 10.3389/fcvm.2022.941142

**Published:** 2022-09-30

**Authors:** Xiangyu Wang, Haiyuan Liu, Shuaipeng Zhang, Shaojun Huang, Chengxin Zhang

**Affiliations:** Department of Cardiovascular Surgery, First Affiliated Hospital of Anhui Medical University, Hefei, China

**Keywords:** cardiac paraganglioma, catecholamine, primary cardiac oncology, neuroendocrine tumor, surgical skill

## Abstract

Cardiac paraganglioma is a kind of rare neuroendocrine tumor characterized by the persistent secretion of catecholamines. Under excessive exposure of catecholamines, some atypical symptoms are presented, including hypertension, arrhythmias, and headache. The case of surgical treatment of a 28-year-old woman with primary cardiac paraganglioma is presented for experience sharing and surgical skill improvements.

## Case presentation

The patient was a 28-year-old woman admitted with the complaint of progressive hypertension complicated with chest pain, tightness, and palpitation for 5 years. In particular, the hypertension of systolic pressure at the 16th week during pregnancy ranging from 110–200 mmHg was managed through combined therapy with labetalol and amlodipine. However, the level of blood pressure was not controlled stably as expected after medical intervention, therefore, the pregnancy termination underwent further treatment of refractory hypertension. Both previous surgical procedures and family genetic disorders were denied.

**Figure 1:**
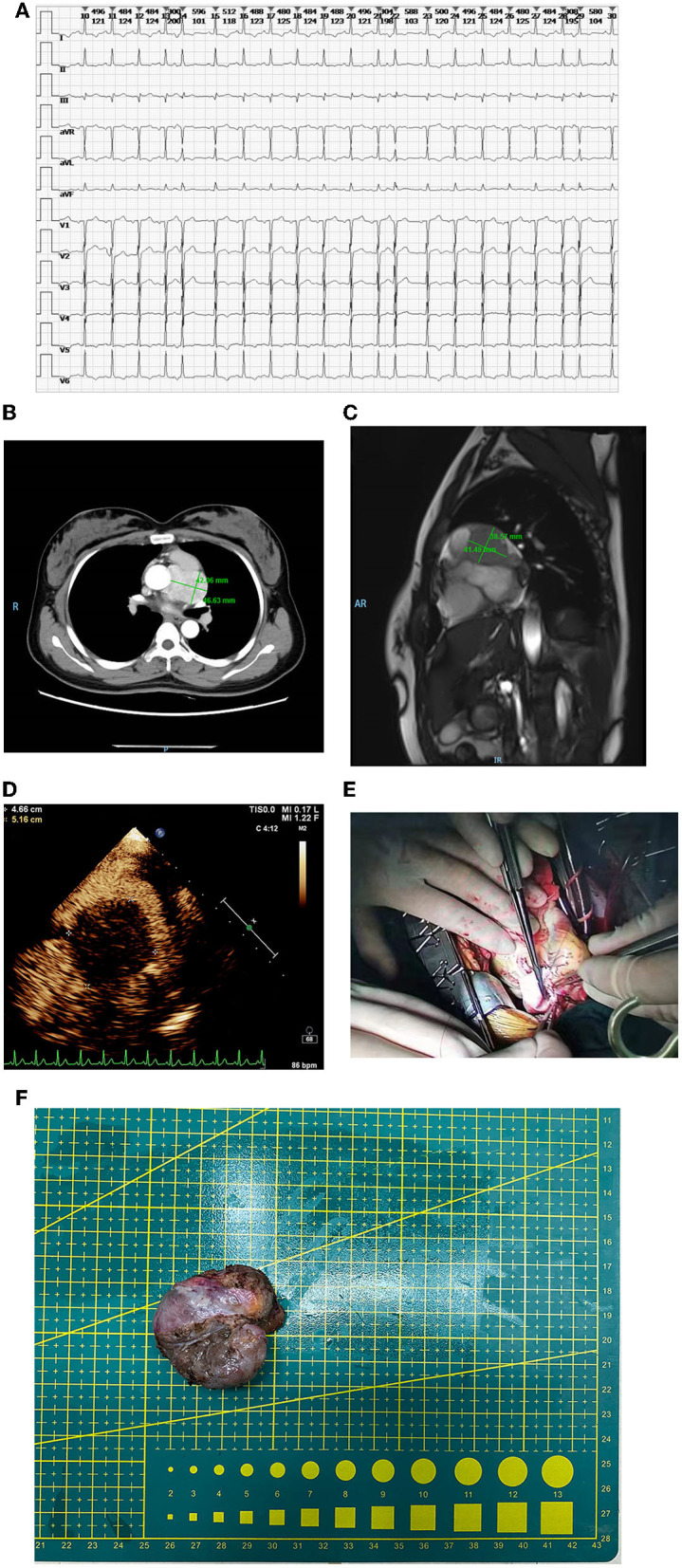
**(A)** Tachycardia in electrocardiogram at admission. **(B)** The mass in the contrast-enhanced CT. The size was 46 × 42 mm and serious adhesion between the aorta and the mass was found. Also, the involved left main coronary was observed. **(C)** The mass in the MRI. Both the root of the aorta and left main coronary were involved, however, the myocardium was free from involvement. **(D)** The mass in the echocardiography. The mass was fully perfused through mini-vascular circulation. **(E)** The repairing of the pulmonary artery. **(F)** The removed mass.

When at admission, tachycardia was found by electrocardiogram (Figure 1A). Based on the findings from the contrast-enhanced CT, it was demonstrated that a mass of 46 × 42 mm with serious adhesion to the surrounding aorta located at the root of the aorta in the middle mediastinum and the left main coronary was also involved (Figure 1B).

In MRI, it was confirmed that both the root of the aorta and left main coronary were involved, however, the myocardium was free from involvement (Figure 1C). In accordance with the echocardiography, the mass was completely perfused through mini-vascular circulation (Figure 1D). After exclusion of adrenal pheochromocytoma with the absence of any abnormal images found in bilateral adrenals, the final diagnosis of primary cardiac paraganglioma was confirmed comprehensively in accordance with the objective results from imaging and lab tests indicating the elevated catecholamine. The α and β receptor blockers were selected as preoperative agents for hypertension control and multidisciplinary consultants were performed, including gynecology and urology for preoperative assessment.

## Surgical procedure

Peripheral cardiopulmonary bypass (CPB) was established *via* the femoral artery and vein. Once CPB started, the median sternotomy was selected. After surgical field exposure, it was found that the mass with the visual size of 65 mm located at the outflow tract of the right ventricle besides the main pulmonary artery was involved seriously. The blood supplement was distributed fully around the mass. Cardiac arrest was maintained using HTK cardioplegia after ascending aorta clamping when the temperature is lower than 35°C. Given the complicated mass-related anatomical changes, extended lesion resection, reconstruction of the pulmonary artery, and coronary artery bypass grafting (CABG) were processed simultaneously. The surgical separation of the mass began at the anterior wall of outflow of the right ventricle. The main pulmonary artery was opened to expose the pulmonary valve due to serious adhesion between the mass and the right ventricle. Then, the separation of the posterior wall of the main pulmonary artery was continued cautiously. Meanwhile, the proximal end of left main coronary was also damaged due to severe involvement by the mass. After the mass was removed completely, a bovine pericardial patch of reasonable size was used to repair the pulmonary artery (Figures 1E,F).

There were two grafts for CABG, great saphenous vein (SVG), and internal mammary artery (LIMA), respectively. First, circumflex (LCX) grafting was performed through end-to-side anastomosis with the proximal end of the great saphenous vein. Then, left anterior descending (LAD) grafting was continued with end-to-side anastomosis with the internal mammary artery. At last, the distal end of the great saphenous vein was sutured at the ascending aorta (AO).

After CABG was completed, regular rewarming was activated and when the temperature was higher than 33°C, the ascending aorta was de-aired and opened. The final closure of the chest was finished after weaning of CPB.

## Postoperative management

**Figure 2:**
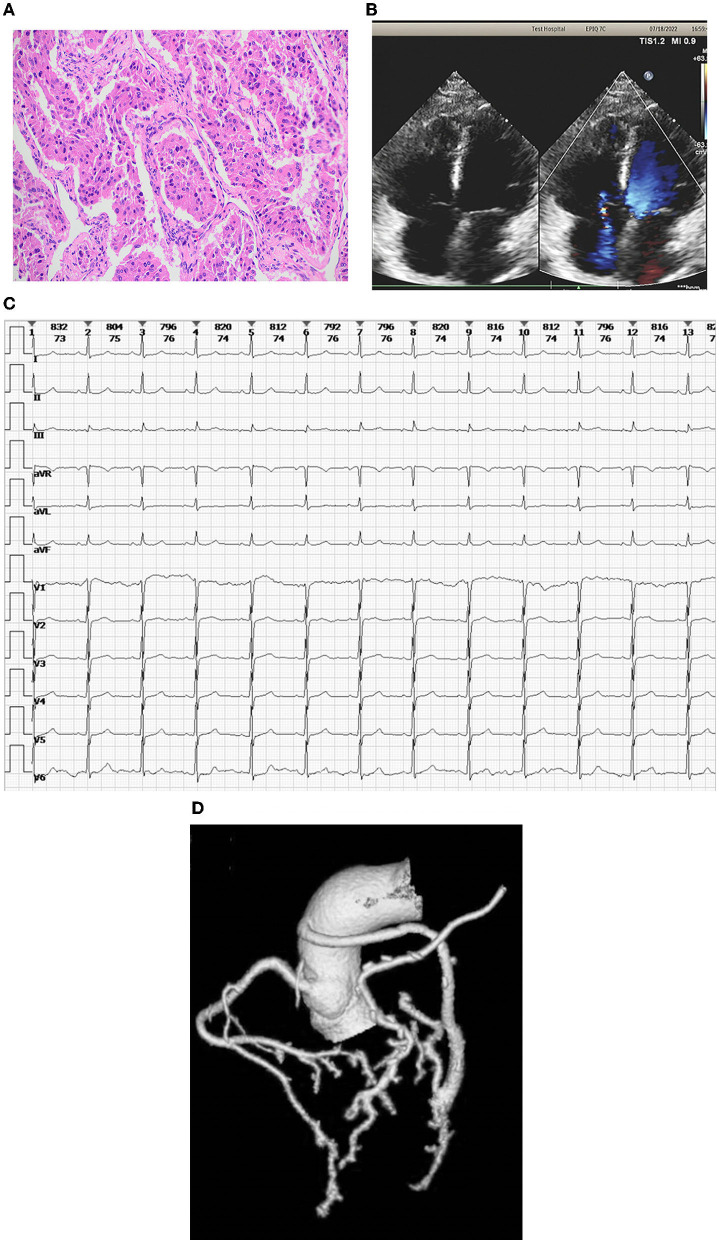
**(A)** Pathology of the removed mass. The pathological findings of the mass were confirmed as cardiac paraganglioma. **(B)** The echocardiography at the latest follow-up. Morphologically, all the chambers and valves were normal and the heart function was stable without any serious abnormal changes. **(C)** Sinus rhythm in electrocardiogram rechecking. **(D)** Coronary CT angiography shows a patent coronary vessel.

The postoperative blood pressure was managed stably and dynamically using α-receptor and β-receptor blockers. Daily administration of vasoactive agents was adjusted and monitored as needed to prevent both organic function and perfusion affected dramatically after surgery. The urine norepinephrine on the 1st day after surgery was 1,953.4 mg/ml. The norepinephrine decreased to 616.7 ng/ml on the 5th day and to the normal level on the 9th day after surgery. The blood pressure was gradually stable and maintained on the 3rd day after surgery. Pathologically, the removed mass was confirmed as cardiac paraganglioma (Figure 2A).

## The outcome and follow-up

The patient was discharged smoothly after clinical assessment where no recurred hypertension, arrhythmias, or any other discomfort complaints were found. During the latest follow-up, it was shown that, morphologically, all chambers and valves were normal and the heart function was stable without any serious abnormal changes (Figure 2B). The sinus rhythm was found by electrocardiogram rechecking (Figure 2C). Furthermore, it was demonstrated *via* the coronary angiography, the patency of grafts for both LIMA–LAD and AO–SVG–LCX was qualified without any obstructions (Figure 2D).

## Discussion

Clinically, the incidence of cardiac paraganglioma is only about 0.6/100,000 (1). All chambers of the heart are potential locating targets for cardiac paraganglioma, however, among them, the left atrial is the predominated candidate (2). It has been demonstrated that genetic mutations are associated with the development of cardiac paraganglioma (3). Moreover, the major mutation is found in the gene SDHB accounting for more than 70% of mutations for cardiac paraganglioma (4). Therefore, it is recommended that all patients with suspected paraganglioma should take a gene test. Unfortunately, this patient refused a gene test due to private reasons. The symptoms of paraganglioma highly depend on the secretion products and the metabolites from tumors, including norepinephrine, epinephrine, and dopamine (5). In principle, the histology of cardiac paraganglioma classified as the extra-adrenal non-epithelial tumor is similar with that of the pheochromocytoma and is mainly considered as a noradrenergic-subtype with the presentation of norepinephrine-related symptoms, such as hypertension, diaphoresis, palpitations, and headache (6). Medically, rather than the secretion level, the location of cardiac paraganglioma is more challenging and significant.

Under persistent secretion of catecholamines, patients with cardiac paraganglioma are more at risk for severe or even fatal complications, including hypertensive crisis, refractory arrhythmias, or myocardial infarction. Hence, as previously reported, both α- and β-adrenoceptor blockers were used for this patient before the surgery to control and adjust the secretion of catecholamines reasonably (7). Moreover, if complicated tachycardia is presented because of the induction by catecholamines, the specific if currency inhibitor, ivabradine is recommended (8). Surgically, the feasibility of cardiac paraganglioma resection should be evaluated cautiously and carefully prior to surgery. The anatomical changes due to the tumor and the structural relationship between the tumor and surrounding organs and tissues are critical in the decision-making of surgical procedures. For our patient, given the history of pregnancy, although underwent pregnancy termination, a differentiated diagnosis for obstetrical complications should also be needed. Besides, it has been found that paraganglioma without any intervention during pregnancy is closely associated with the high incidence of complications for both pregnant patients and fetuses. Therefore, earlier diagnosis and medical intervention during pregnancy are positive and valuable in improving outcomes and survival for both of them and the most suitable period for surgical resection is no longer than the 24th week of gestation (9, 10). During the surgery, surgical resection was processed under the assistance of CPB established with the peripheral femoral artery-vein path aiming to keep enough space for anastomosis due to serious involvement of the posterior wall of the main pulmonary artery. Following the mass resection, vascular reconstruction and repairing are performed with autologous, xenogeneic, and synthetic materials. In addition, it should be emphasized that the left main coronary of this patient was involved in cardiac paraganglioma, also, CABG is required to reconstruct the system of the coronary artery. Both SVG and LIMA, are the main prepared grafts (11).

Postoperation, management of blood pressure and organic perfusion during the transition period with the absence of excessive stimulation under catecholamines and prevention of recurrence of the tumor are major points influencing the outcome. The improvement for both postoperative survival and life quality is significant and qualified as long as no metastatic lesions are found (12). During the postoperative follow-up of 6 months, the recurred tumor and metastatic lesions or any other tumor-related discomforts and complaints have not been found.

Furthermore, the therapies for patients with metastatic paraganglioma are summarized as chemotherapy, radiotherapy, targeted therapy, and combination therapy. For instance, the combination therapy of cyclophosphamide, vincristine, and dacarbazine following the temozolomide monotherapy is considered the first-line conventional strategy for patients with progressive paraganglioma complicated with SDHB mutation (13). Also, Sunitinib is proved to be the indication of therapy for patients with progressive or even inoperable paraganglioma (14). Clinically, for this kind of rare and complicated tumor and in accordance with various different involved organs and/or tissues, a multidisciplinary team can be assembled to provide more suitable and optimal options in decision-making for postoperative management and dynamic observation during long-term follow-up (15).

## Conclusion

Though it is uncommon, the symptoms and complications related to cardiac paraganglioma are severe and even life-threatening, especially for pregnant female patients. Surgical treatment is the most effective strategy against cardiac paraganglioma, nevertheless, the difficulty and feasibility of mass resection should be assessed fully before surgery. During the surgical procedures, completed mass resection and necessary tissue reconstruction are required and CABG should be potential as needed. Periodical follow-up after surgery is useful to evaluate any recurrence or metastasis. If necessary, further medical intervention, such as chemotherapy, radiotherapy, and targeted therapy, may be continued.

## Data availability statement

The original contributions presented in the study are included in the article/supplementary material, further inquiries can be directed to the corresponding author/s.

## Ethics statement

Written informed consent was obtained from the patient for the publication of this case report and any accompanying images and data.

## Author contributions

All authors listed have made a substantial, direct, and intellectual contribution to the work and approved it for publication.

## Conflict of interest

The authors declare that the research was conducted in the absence of any commercial or financial relationships that could be construed as a potential conflict of interest.

## Publisher's note

All claims expressed in this article are solely those of the authors and do not necessarily represent those of their affiliated organizations, or those of the publisher, the editors and the reviewers. Any product that may be evaluated in this article, or claim that may be made by its manufacturer, is not guaranteed or endorsed by the publisher.
